# Regret, uncertainty, and bounded rationality in norm-driven decisions

**DOI:** 10.1371/journal.pone.0354748

**Published:** 2026-08-07

**Authors:** Christos Charalambous

**Affiliations:** Department of Economics, University of Cyprus, Nicosia, Cyprus; Georg-August-Universität Göttingen: Georg-August-Universitat Gottingen, GERMANY

## Abstract

This study introduces an agent-based model to examine how regret, uncertainty, and social norms interact to shape vaccination behavior during epidemics. The model integrates three behavioral mechanisms—anticipated regret, evolving norms, and uncertainty-dependent trust—within a unified learning framework. Grounded in psychology and behavioral economics, it captures how individuals make probabilistic choices influenced by material payoffs, fear, trust, and social approval. Simulations of the Susceptible–Infected–Recovered process show that choice precision has context-dependent effects. In the learning-only baseline, collective outcomes are best at intermediate precision, where agents respond sufficiently to risk while remaining flexible enough to adapt. When social norms are included, this relationship changes: normative feedback stabilizes behavior and reduces the cost of highly precise choices, while high decision noise remains harmful. Regret exerts a dual influence—moderate levels encourage adaptive self-correction, while excessive regret or greed destabilize choices. Uncertainty has a similarly non-linear effect: moderate ambiguity promotes caution, but too much uncertainty disrupts coordination. Social norms restore cooperation by compensating for incomplete information. Personal norms guide behavior when individuals have reliable information and feel confident in their judgments. Injunctive norms—signals of others’ approval—become more influential under uncertainty, while descriptive norms, which arise from observing others’ actions, provide informational cues that help people decide what to do when direct knowledge is limited. Overall, the model provides a psychologically grounded, computationally explicit account of how emotion, cognition, and social norms jointly govern preventive behavior during epidemics.

## 1. Introduction

The persistence of vaccine hesitancy during recent epidemics shows that preventive behavior cannot be explained by instrumental rationality alone. Vaccination decisions arise from an interplay of material incentives, emotions, social expectations, and uncertainty about both disease dynamics and social reactions. Traditional epidemic and imitation-based models capture strategic and diffusion aspects but often overlook the psychological and normative forces driving real-world responses [1,2]. Understanding how these forces interact is crucial for anticipating compliance and designing effective interventions.

Social norms—people’s perceptions of what others do (descriptive norms) and what others approve of (injunctive norms)—strongly shape vaccination behavior [1,3,4]. Descriptive norms guide behavior through informational cues and are most influential under uncertainty about others’ actions, whereas injunctive norms operate via moral and reputational channels [5]. Aligning these two norms enhances compliance, while misalignment undermines it [2,6]. Large-scale studies show that providing accurate normative information—such as evidence of high vaccination rates—increases willingness to vaccinate, particularly among uncertain or mistrustful individuals [7]. Experimental evidence from “pandemic-like” environments also shows that individuals respond very differently to descriptive and injunctive cues; in particular, Woike et al. [8] found that only injunctive messages consistently reduced risk-taking, while descriptive messages could even backfire by increasing it. These findings underscore the importance of treating social norms as multidimensional influences rather than uniform informational signals, highlighting the need for models that capture how descriptive and injunctive expectations coevolve.

Emotions are equally central. Regret theory [9] shows that individuals evaluate outcomes relative to forgone alternatives, incorporating counterfactual emotions into choice. Anticipated regret increases vaccination uptake by raising the emotional cost of inaction [10,11], while worry mediates the link between perceived risk and compliance [12,13]. Thus, emotional forecasting and normative expectations jointly shape preventive behavior, motivating an integrated modeling approach.

Uncertainty compounds these processes. Individuals rarely know others’ infection or vaccination status, nor the reliability of their information sources. This imperfect information translates into subjective uncertainty—a lack of confidence in epidemiological and social beliefs. Empirical evidence shows that uncertainty—whether arising from incomplete social visibility or from low confidence in risk judgments—amplifies imitation and descriptive conformity while weakening injunctive guidance [14]. Emotional arousal and unstable trust further modulate these effects [4,15]. Under uncertainty, decision weights shift dynamically across material, emotional, and normative domains. Our model formalizes this process through uncertainty- and fear-dependent weighting functions that bridge informational and psychological traditions [12,16].

Agent-based modeling (ABM) offers the ideal framework to integrate these mechanisms. ABMs represent heterogeneous, boundedly rational agents with emotional and social biases [17,18] interacting on structured networks [19]. They reveal how local mechanisms—regret, conformity, trust—aggregate into collective equilibria and how feedback among emotion, information, and network structure drives norm evolution [20,21]. In vaccination contexts, ABMs have examined how social norms and perceived risks coevolve [18] and how learning rules affect uptake [22]. Combining behavioral experiments with simulation helps identify when societies transition between cooperation and defection [21].

A substantial body of work has used agent-based models to study vaccination behavior and other preventive responses under bounded rationality, social influence, and heterogeneous risk perception. These models have shown that imitation, social learning, and local interaction structure can strongly shape both vaccination uptake and epidemic outcomes [22–24]. Related work has also emphasized the role of social norms and behavioral feedback, showing that vaccination decisions are shaped not only by material incentives but also by expectations about others’ behavior and by broader processes of norm formation and compliance [1,18,21,25]. Other psychologically informed models have incorporated reinforcement learning, probabilistic choice, and context-dependent adaptation, thereby moving beyond purely payoff-maximizing formulations of epidemic behavior [26–28].

At the same time, these lines of research have usually been developed separately. Models of vaccination behavior often include learning, imitation, and local social influence, but they typically do not distinguish between personal norms, descriptive expectations, and injunctive expectations [22,23]. Models of norm dynamics make this distinction more explicit, but they are rarely placed in a repeated epidemic setting where agents learn from infection outcomes and update regret-adjusted payoffs over time [21,28]. Conversely, regret theory explains how people evaluate choices by comparing actual or anticipated outcomes with forgone alternatives [9,29,30], but this mechanism is usually studied outside a coevolving social-norm environment. As a result, we still know relatively little about how regret, uncertainty, and different forms of normative influence behave when they operate together in the same adaptive epidemic process.

The present paper addresses this gap by developing an agent-based behavioral epidemic model that combines these mechanisms in a single framework. Agents evaluate vaccination through material payoffs, regret-adjusted feedback, and three norm components: personal norms, descriptive expectations, and injunctive expectations. Their choices are probabilistic, following a logit-type rule commonly used to represent boundedly rational choice under uncertainty [26,31]. The weight assigned to each decision input changes with perceived safety, uncertainty, trust, and local social stability. This is motivated by evidence that emotions and perceived risk shape preventive behavior [11,32], and that low trust or incomplete information can increase reliance on social cues [7]. The model therefore links payoff-based learning, emotional feedback, and evolving social expectations within a repeated decision process.

We use this framework to address three questions. First, how do regret and uncertainty jointly shape vaccination decisions and epidemic outcomes? Second, when do evolving social norms improve cooperation? Third, how does choice precision affect the balance between adaptive self-correction and instability? The aim is not to restate the empirical regularities that motivate the model, but to examine what follows when they interact over repeated epidemic seasons. The simulations show that these interactions generate system-level patterns: choice precision can have non-monotonic effects, regret can either support self-correction or destabilize behavior depending on choice precision, and uncertainty can change which norm-based interventions are most effective. These outcomes are interpreted as emergent properties of the coupled system, in line with the agent-based modeling view that aggregate patterns arise from interactions among micro-level mechanisms rather than being imposed directly at the population level [20,21].

## 2. Model

During a pandemic, two coupled processes unfold in parallel: the epidemiological spread of infection and the behavioral adaptation of individuals responding to it. Because each shapes the other, they must be modeled jointly. Disease transmission occurs on a physical-contact network following the classical Susceptible–Infected–Recovered (SIR) framework, while behavioral adaptation evolves on a social network where individuals observe and influence one another.

**Fig 1:**
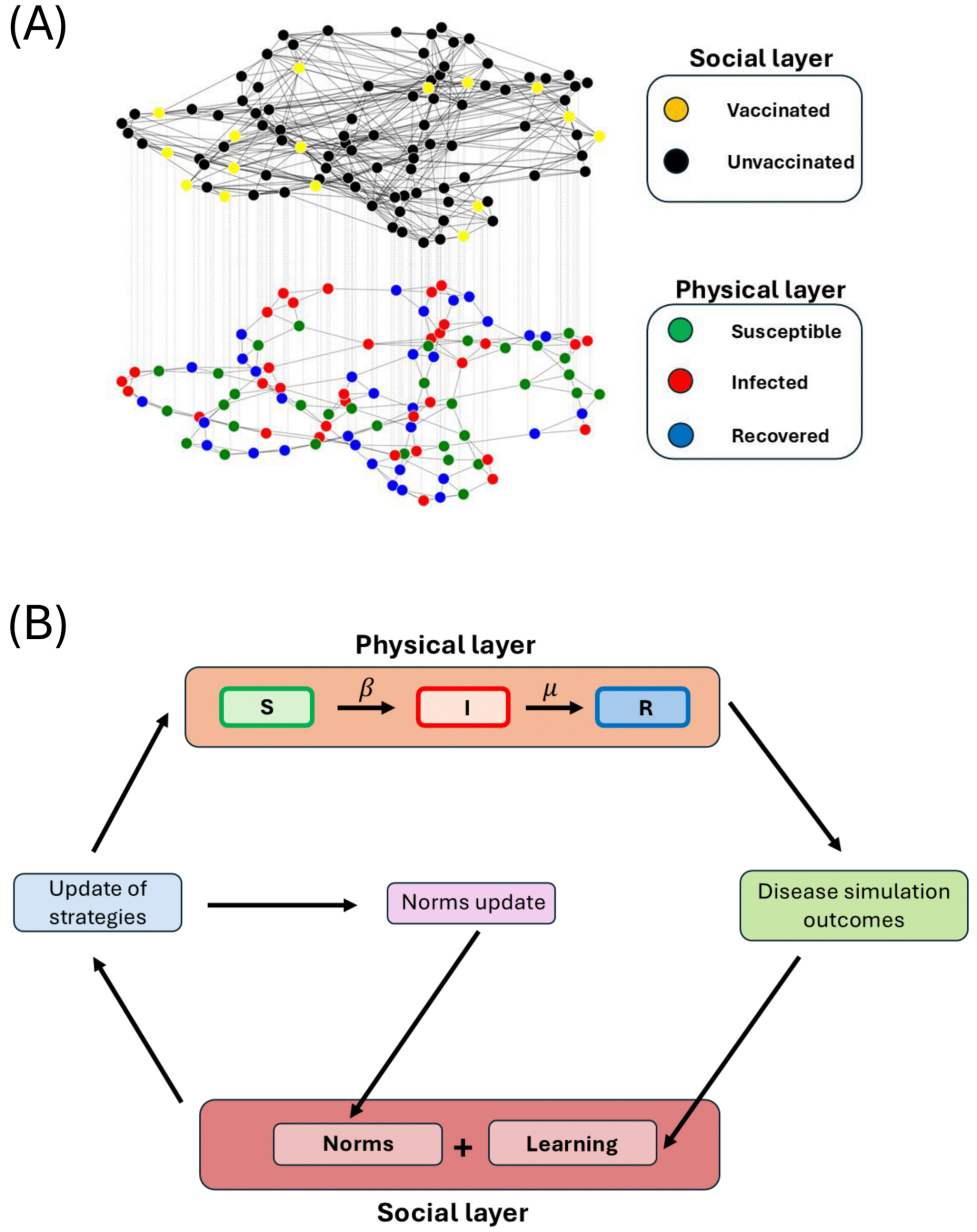
Left: Algorithm of system’s dynamics. The flowchart outlines the sequence of processes within each epidemic season. The model first simulates infection dynamics on the physical layer, estimating each agent’s infection probability. After each season, agents decide whether to vaccinate based on the previous outcomes, integrating both material (learning-based) and normative considerations. Subsequently, they update their norms before the next season begins. **Right (A): Schematic of the two layer multiplex network.** The model operates on two interconnected layers. On the *physical layer*, infection propagates through a Susceptible–Infected–Recovered (SIR) process; on the *social layer*, agents observe outcomes and peers’ actions to guide vaccination. The physical layer follows a small-world topology, while the social layer uses a Klimek–Thurner structure—both empirically supported as realistic for contact and communication networks. A significant overlap between layers reflects that many physical ties also transmit social information. **(B): Dynamics of the vaccination game.** Schematic of the algorithm. Each season, the SIR dynamics run on the physical layer to estimate infection probabilities. Agents then decide whether to vaccinate, considering both payoff-based learning and normative factors, update their norms accordingly, and begin the next season.

Although network topology is not the primary focus, we employ empirically grounded synthetic structures (Fig 1). The physical layer $G^{Phys}$ follows a small-world network [33] with average degree 6 and rewiring probability 0.1, and the social layer $G^{Soc}$ is generated using the Klimek–Thurner model [34] (with parameters *r* = 0.12, *c* = 0.58 and *m* = 1). To reflect that many physical interactions also convey information, we assume partial overlap between the two layers. Tests with alternative topologies, such as Erdős–Rényi graphs, yield qualitatively consistent results.

At the start of each season, agents decide whether to vaccinate, and one individual is initialized as infected. The process repeats across seasons until equilibrium is reached. Within each season, multiple SIR realizations are simulated using an event-driven algorithm [35] to estimate infection probabilities. The transmission and recovery rates are denoted by β and μ; since μ only determines timescale, it is normalized to μ=1.

Behavioral adaptation is modeled game-theoretically, as each individual’s payoff depends on others’ choices. Vaccination decisions are influenced not only by perceived disease risk but also by beliefs and social norms. Following [28], each individual *i* possesses three normative components: (i) a personal attitude $y_{i}$ reflecting moral preference; (ii) an empirical expectation ${\tilde{x}}_{i}$ representing perceived peer behavior; and (iii) a normative expectation ${\tilde{y}}_{i}$ capturing perceived collective approval.

Focusing on seasonal epidemics, we integrate information from repeated SIR simulations with agents’ evolving norms to determine vaccination choices at each new season. Rather than continuously tracking norm change, we update all normative variables only at decision points—the start of each season—capturing how experience and feedback shape subsequent behavior. The complete algorithmic structure of this coupled epidemiological–behavioral system is illustrated in Fig 1.

### 2.1. Model assumptions

The model rests on the following assumptions.

1. The system evolves on two partially overlapping networks: a physical-contact layer on which infection spreads, and a social layer on which agents observe and influence one another [33–35].
2. Time is discrete at the behavioral level and proceeds in epidemic “seasons.” At the start of each season, agents decide whether to vaccinate; within the season, infection spreads according to an SIR process [35,36].
3. Agents do not observe the infection states of all neighbors. Instead, each agent observes only a fraction of local states, which gives rise to informational uncertainty [12,16].
4. Vaccination decisions reflect four components: material payoff, personal norm, descriptive expectation, and injunctive expectation [1,28,37].
5. The relative weights assigned to these components are adaptive and depend on perceived safety, uncertainty, peer stability, and local consensus [14,15,38].
6. Regret enters through counterfactual comparison of the two available actions, while realized outcomes from previous seasons influence future regret-adjusted valuations through learning [9,29,36].
7. Personal norms, injunctive expectations, and descriptive expectations evolve at different rates, with personal norms assumed to change most slowly [28,38,39].
8. The payoff from vaccination is represented as a fixed direct cost, whereas the payoff from non-vaccination depends on realized or expected infection outcomes and is therefore uncertain [13,36].
9. Choice follows a probabilistic logit (Fermi-type) rule, so agents respond to utility differences with finite precision rather than in a fully deterministic manner [26,31,40].
10. Initial norm values are drawn from broad distributions in order to represent heterogeneous and weakly structured initial conditions rather than a calibrated empirical population.

**Algorithm 1. Evolutionary Update Rule.**

1:  **Input:** Networks $G^{Phys}$, $G^{Soc}$, SIR (β,μ), Utility $\left(c_{V},c_{I},\kappa ,m,\eta_{1},\eta_{2}\right)$, Norms $\left(y_{i},{\tilde{x}}_{i},{\tilde{y}}_{i}\right)$, stopping tolerance ϵ

2:  **Output:** *I*(*t*), $x_{i}(t)$, $\left(y_{i},{\tilde{x}}_{i},{\tilde{y}}_{i}\right)$

3:  Initialize norms and SIR state.

4:  **for** season t=0,1,2,... **do**

5:   Run $n_{sim}$ SIR realizations; compute $E_{i}[P](t)$, $S_{i}(t)$, $f_{i}(t)$, ${\hat{I}}_{i}(t)$.

6:   Learning: update ${\hat{\pi }}_{i}^{\text{Unvac}}(t)$ via Eq. (19); set ${\hat{\pi }}_{i}^{\text{Vac}}=1-c_{V}$.

7:   Regret (LS): $R(x)=\eta_{1}x^{\eta_{2}}$ if *x* > 0 else 0; adjust $\pi_{i}^{\text{Vac}},\pi_{i}^{\text{Unvac}}$.

8:   Weights: compute $u_{i},q_{i}^{\text{stab}}$, $q_{i}^{\text{cons}}$; then $A_{\text{mat}},A_{y},A_{\tilde{x}},A_{\tilde{y}}$.

9:   Utilities: $\Delta U_{i}$ from Eq. (4); intention $x_{i}=(1+e^{-\Delta U_{i}/\kappa }{)}^{-1}$; draw $a_{i}$.

10:  Norm dynamics: update $\left(y_{i},{\tilde{y}}_{i},{\tilde{x}}_{i}\right)$ via Eqs. (22).

11:  If $|\langle x_{i}\rangle_{t+1}-\langle x_{i}\rangle_{t}|<\varepsilon$ for 50 seasons: **break**.

12: **end for**

### 2.2. Initialization and simulation procedure

At the beginning of each simulation run, personal norms, descriptive expectations, and injunctive expectations are initialized independently from broad distributions, representing heterogeneous and only weakly structured initial conditions. Unless stated otherwise, no agent is initially vaccinated and one individual is selected as the initial infected case. In each behavioral season, the model runs repeated SIR realizations on the physical layer in order to estimate the infection-related quantities that enter subsequent decisions [35,36]. Agents then update their vaccination intention and action, after which normative variables are updated before the next season begins. Simulations continue until the system reaches an approximate behavioral equilibrium, defined by a sufficiently small change in average vaccination intention over a sustained window of seasons.

### 2.3. Decision making process

To study vaccination intentions, we first define the utility associated with each available action. The *Regret–Uncertainty model* combines three empirically grounded mechanisms—anticipated regret, social norms, and uncertainty-dependent trust—within a single decision framework. Preventive behavior is thus shaped not only by instrumental payoffs but also by affective and social considerations, especially under uncertainty and interdependence [1,9,14].

Let a∈{0,1} denote the action, where *a* = 0 corresponds to not vaccinating and *a* = 1 to vaccinating. The action-specific utility of agent *i* is given by


$$\begin{array}{c} U_{i}(\pi_{Vac},\pi_{Unvac},a,y,\tilde{x},\tilde{y})=A_{mat}\;\Pi_{i}(\pi_{Vac},\pi_{Unvac},a)+V_{i}(a,y,\tilde{x},\tilde{y}), \end{array}$$ (1)

where a∈{0,1} represents the action (0 = not vaccinate, 1 = vaccinate), *y* is the personal norm, and $\tilde{x},\tilde{y}$ are the empirical and normative expectations respectively. The first term captures material considerations weighted by $A_{mat}$; the second term reflects psychological and social influences.

**Material utility:**


$$\begin{array}{c} \Pi_{i}(\pi_{Vac},\pi_{Unvac},a)=a\pi_{Vac}+(1-a)\pi_{Unvac}, \end{array}$$ (2)

where $\pi_{Vac}$ and $\pi_{Unvac}$ are average payoffs from vaccinating or not over the last *m* seasons.

**Normative utility** [41]:


$$\begin{array}{c} V_{i}(a,y,\tilde{x},\tilde{y})=A_{y}|a-y|+k_{a}|1-a-\tilde{x}|-k_{d}|a-\tilde{x}|+A_{\tilde{y}}(2\tilde{y}-1)(a-0.5), \end{array}$$ (3)

where $A_{y},k_{a},k_{d},$ and $A_{\tilde{y}}$ quantify the strengths of:

1. **Cognitive dissonance**: mismatch between behavior and moral belief $A_{y}|a-y|$;
2. **Descriptive conformity**: alignment with peers’ actions $k_{a}|1-a-\tilde{x}|-k_{d}|a-\tilde{x}|$; Parameters $k_{a}$ and $k_{d}$ characterize the psychic benefit of approval and the costs of disapproval by peers, respectively, as in [41].
3. **Injunctive conformity**: sensitivity to perceived moral approval $A_{\tilde{y}}(2\tilde{y}\;-\;1)(a\;-\;0.5)$

This formulation integrates cognitive dissonance and social conformity into a single additive structure, reflecting how moral coherence and peer alignment jointly shape utility.

Choice depends not on the utility of one action considered in isolation, but on the difference between the utilities of vaccinating and not vaccinating. Following [41], this difference can be written as:


$$\begin{array}{c} \Delta U_{i}=U_{i}(1)-U_{i}(0)=A\;\left[\frac{2}{A}\;\left(A_{mat}p_{learn}+A_{y}y+A_{\tilde{x}}\tilde{x}+A_{\tilde{y}}\tilde{y}\right)-1\right], \end{array}$$ (4)

where


$$\begin{array}{c} p_{learn}=0.5+0.5(\pi_{Vac}-\pi_{Unvac}),\;A=A_{mat}+A_{y}+A_{\tilde{x}}+A_{\tilde{y}}. \end{array}$$ (5)

#### 2.3.1. Probabilistic choice and bounded rationality.

The probability that agent *i* vaccinates follows a Fermi-type response function:


$$\begin{array}{c} x_{i}=\frac{1}{1+e^{-\Delta U_{i}/k_{rat}}}, \end{array}$$ (6)

where $k_{rat}$ is an inverse choice-precision parameter. Small values of $k_{rat}$ imply that agents respond more deterministically to utility differences, whereas large values imply noisier and less utility-sensitive choice. In this sense, $k_{rat}$ should not be interpreted as “rationality” in a broad cognitive or philosophical sense, but rather as the precision with which agents translate perceived utility differences into behavior. At the same time, the broader framework is naturally described as one of bounded rationality, since the Fermi-type specification is a standard logit or quantal-response rule used to represent probabilistic departures from perfect optimization under finite precision [26,31,40]. Without loss of generality, we set *A* = 1. The coefficients $A_{mat}$, $A_{y}$, $A_{\tilde{x}}$, and $A_{\tilde{y}}$ denote the adaptive weights of material, personal, descriptive, and injunctive components, respectively, whose evolution is described below.

#### 2.3.2. Dynamical weights.

To determine how each component contributes to decision-making, we rely on empirical findings linking normative influence to psychological states and environmental stability.

Studies show that as infection risk increases, individuals rely more on descriptive than injunctive norms [42], and uncertainty about susceptibility amplifies the motivational power of descriptive norms [14]. Under fear, people seek more social information [43], and perceived knowledge insufficiency fosters imitation and peer reliance [12]. However, such dependence presupposes that peers are stable and relatively consistent in their behavior.

Integrating these findings, we define:


$$\begin{array}{ccc} A_{mat}(\phi_{i}^{Emp},\phi_{i}^{Col}) & = & \phi_{i}^{Emp}(1-\phi_{i}^{Col}), \end{array}$$ (7)

$$\begin{array}{ccc} A_{y}(\phi_{i}^{Emp},\theta_{i}^{Col}) & = & (1-\phi_{i}^{Emp})(1-\theta_{i}^{Col}), \end{array}$$ (8)

$$\begin{array}{ccc} k_{a}+k_{d}=A_{\tilde{x}}(\phi_{i}^{Emp},\phi_{i}^{Col}) & = & \phi_{i}^{Emp}\phi_{i}^{Col}, \end{array}$$ (9)

$$\begin{array}{ccc} A_{\tilde{y}}(\phi_{i}^{Emp},\theta_{i}^{Col}) & = & (1-\phi_{i}^{Emp})\theta_{i}^{Col}, \end{array}$$ (10)

which satisfy *A* = 1 and $A_{j}\in [0,1]$ for all *j*. In addition, $k_{a}+k_{d}$ can be viewed as a measure of the strength of injunctive social norms. Here, $\phi_{i}^{Emp}$ balances empirical and injunctive influence, while $\phi_{i}^{Col}$ and $\theta_{i}^{Col}$ (the *trust coefficients*) govern the weight assigned to collective versus individual cues:


$$\begin{array}{c} \phi_{i}^{Emp}=(f_{i}u_{i}{)}^{1/2},\;\phi_{i}^{Col}=(q_{i}^{stab}q_{i}^{cons}f_{i}u_{i}{)}^{1/4},\;\theta_{i}^{Col}=(q_{i}^{stab}q_{i}^{cons}f_{i}u_{i}^{intr}{)}^{1/4}. \end{array}$$ (11)

Here $f_{i}=1-S_{i}(t)$ quantifies fear, increasing as perceived safety $S_{i}(t)$ decreases:


$$\begin{array}{c} S_{i}(t)=\left\{ \begin{array}{cc} 1-{\hat{I}}_{i}(t), & \text{if unvaccinated},\\ 1-E_{i}[P](t), & \text{if vaccinated}, \end{array}\right. \end{array}$$ (12)

where ${\hat{I}}_{i}(t)=n_{i,sim}^{inf}(t)/n_{sim}$, with $n_{sim}$ the total number of simulations and $n_{i,sim}^{inf}(t)$ the total number of simulations in which the $i^{th}$ agent was infected, while $E_{i}[P](t)$ is the expected infection probability derived from infected neighbors, which will be defined in the next subsection.

Total uncertainty is


$$\begin{array}{c} u_{i}=u_{i}^{intr}+u_{i}^{info}, \end{array}$$ (13)

where $u_{i}^{intr}$ represents intrinsic uncertainty (lack of confidence) and $u_{i}^{info}$ captures informational uncertainty arising from incomplete knowledge of neighbors’ infection states.

The variable $q_{i}^{stab}$ quantifies peer stability based on the change-detector function [44]:


$$\begin{array}{c} q_{i}^{stab}(t)=1-\frac{1}{2}Q_{i}(t),\;Q_{i}(t)=\sum_{k=1}^{m-1}(h_{i}^{k}(t)-q_{i}^{k}(t)), \end{array}$$

where $h_{i}^{k}(t)$ measures historical frequencies of neighbor actions over a memory window *m*, and $q_{i}^{k}(t)$ records current strategies. High volatility (large $Q_{i}$) decreases $q_{i}^{stab}$, prompting agents to rely more on self-learning.

Local consensus is given by $q_{i}^{cons}=2|X_{i}-0.5|$, where $X_{i}$ is the fraction of vaccinated neighbors. Thus, $q_{i}^{cons}=0$ when opinions are evenly split and $q_{i}^{cons}=1$ when full agreement exists.

The equations define the weights as geometric means of fear, trust, and uncertainty—an appropriate formulation when independent factors jointly determine the reliability of social information. This multiplicative rule ensures that if any component is weak, overall influence declines sharply, consistent with evidence that (i) fear without trust leads to disengagement [45], (ii) trust without fear lacks motivational urgency [12], and (iii) uncertainty without credible input results in inaction.

The functional forms in Eqs. (7)–(11) should therefore be read as a parsimonious structural hypothesis rather than as a calibrated measurement model. Their purpose is to encode a simple but substantively meaningful idea: social influence should be strongest when agents are simultaneously uncertain, attentive to risk, and embedded in a local environment that is sufficiently stable and coherent to be informative. A multiplicative specification is a natural way to capture this complementarity, because it prevents any one component from dominating when the others are weak. The qualitative results should thus be interpreted primarily at the level of this joint-dependence logic, rather than as depending on one exact parametric form.

**Fig 2:**
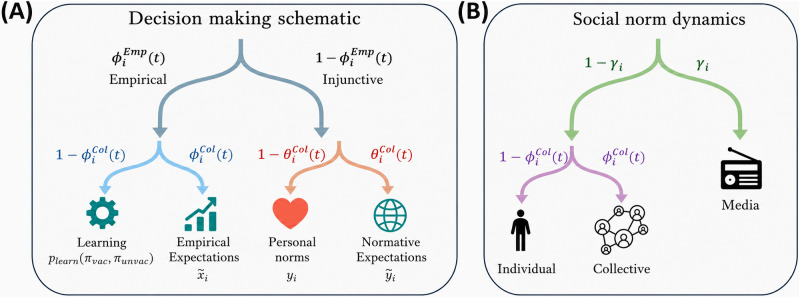
(A) Schematic of the decision-making process. The diagram illustrates how learning and social norms jointly determine each agent’s vaccination intention, weighted by their relative influence. Two assumptions guide this interaction: (a) The less safe an agent feels—i.e., the lower $S_{i}(t)$, representing infection risk from the previous season—the more they rely on empirical cues (learning or empirical expectations) rather than normative ones (personal beliefs or injunctive expectations). Similarly, the more uncertain the agent feels about the knowledge he posses, the more he will consult his injunctive, moral variables (personal and normative expectations). (b) The more heterogeneous and unstable the environment—when agents’ recent decisions diverge from one another or from past averages—the greater the reliance on personal experience and beliefs instead of social imitation. **(B): Schematic of the dynamics of social norm variables.** The diagram shows how different factors shape norm evolution under two key assumptions: (a) Each agent updates her norms with probability $\gamma_{i}$ under external influence, otherwise combining personal beliefs, existing norms, and peers’ actions. (b) Environmental instability—large deviations between recent and past behaviors and low consensus among neighbors—increases dependence on personal factors over conformity to others.

By combining these drivers multiplicatively, the model captures how individuals integrate emotional, cognitive, and social cues under risk. Fear heightens perceived vulnerability, trust legitimizes information, and uncertainty fosters openness to influence. Together, these mechanisms determine how descriptive and injunctive norms guide vaccination behavior. The overall decision process is summarized schematically in Fig 2.

The distinction between empirical and normative inputs should be understood as a distinction in decision weight rather than as a claim that norms are somehow non-empirical in origin. The idea is that under elevated perceived risk, agents give more weight to cues that appear directly relevant to immediate consequences, such as recent behavioral and epidemiological information [12,14]. When perceived danger is lower, by contrast, social alignment and moral consistency can play a relatively larger role in shaping action [1,38]. The model therefore does not treat norms as detached from experience. Instead, it assumes that different forms of information become more or less salient as the perceived risk environment changes.

#### 2.3.3. Uncertainty.

During COVID-19, countries adopted markedly different digital contact-tracing systems. Centralized platforms such as China’s *Alipay Health Code* and South Korea’s *Corona 100 m* relied on government databases to provide real-time information about contacts with infected, non-infected, or untested individuals [46–50]. In contrast, decentralized approaches like the MIT-led *SafePaths* project stored contact histories locally and compared them with anonymized case data, alerting users to potential exposure without revealing identities [51–56]. Although such systems typically omitted encounters with non-infected or untested persons, expanding their functionality to include these would be technically feasible.

Building on this context, we model epidemic dynamics under partial observability of the social network. Rather than assuming that individuals know the infection status of all contacts, we posit that each agent observes only a subset of neighbors—reflecting the limited information available through digital tracing tools. This incomplete visibility introduces uncertainty about true infection risk. The repeated SIR realizations used here are the same internal epidemic simulations that drive the seasonal dynamics of the model [35]. They are not intended to reproduce a specific real-world epidemic dataset, but rather to provide stylized and internally consistent estimates of the infection-related quantities that agents use when updating behavior.

To quantify informational uncertainty, we characterize each agent’s perceived infection risk and the ambiguity surrounding this assessment. Specifically, for each agent *i*, we compute the expected infection probability $E_{i}[P]$, which represents the agent’s perceived risk of becoming infected, and the corresponding variance ${Var}_{i}[P]$, which captures the uncertainty in this perception arising from incomplete information about neighbors’ infection states. Let $n_{i}^{\text{total}}$ denote the total number of neighbors of agent *i*. We assume that only a fraction z∈[0,1] of these neighbors are observable, so that the number of observed neighbors is $n_{i}^{\text{obs}}=z\;n_{i}^{\text{total}}$, while the remaining neighbors are unobserved. Using repeated SIR simulations, we estimate the average fraction of infected neighbors of agent *i* at season *t* as


$$\begin{array}{c} {\hat{I}}_{i,\text{neighbors}}(t)=\frac{n_{\text{sim}}^{\text{inf,neigh}}(t)}{n_{\text{sim}}}, \end{array}$$ (14)

where $n_{\text{sim}}^{\text{inf,neigh}}(t)$ is the total number of simulation runs in which a given neighbor of *i* is infected, and *n*_sim_ is the total number of runs. Among the observed neighbors, the expected number of infected contacts is therefore


$$\begin{array}{c} k_{i}^{\text{obs}}=n_{i}^{\text{obs}}\;{\hat{I}}_{i,\text{neighbors}}(t), \end{array}$$ (15)

which represents the average number of infected neighbors whose states are known to agent *i*.

The remaining $n_{i}^{\text{unobs}}=n_{i}^{\text{total}}-n_{i}^{\text{obs}}$ neighbors have unknown states and are assumed independently infected with probability *p*. The number of infected unobserved neighbors then follows


$$\begin{array}{c} X\sim Binomial(n_{i}^{\text{unobs}},p). \end{array}$$

For a realization *x* of *X*, the total number of infected contacts is $k=k_{i}^{\text{obs}}+x$. Assuming a per-contact transmission probability $\beta =1-e^{-\lambda }$, the infection probability conditional on *k* infected contacts is


$$\begin{array}{c} P_{k}=1-(1-\beta {)}^{k}. \end{array}$$

The expected infection probability is


$$\begin{array}{c} E_{i}[P]=\sum_{x=0}^{n_{i}^{\text{unobs}}}\mathrm{Pr}(X=x)\;[1-(1-\beta {)}^{k_{i}^{\text{obs}}+x}], \end{array}$$

where Pr(X=x) is the corresponding binomial probability. The second moment and variance are


$$\begin{array}{c} E_{i}[P^{2}]=\sum_{x=0}^{n_{i}^{\text{unobs}}}\mathrm{Pr}(X=x)\;[1-(1-\beta {)}^{k_{i}^{\text{obs}}+x}{]}^{2},\;{Var}_{i}[P]=E_{i}[P^{2}]-(E_{i}[P]{)}^{2}. \end{array}$$

We define the informational uncertainty of susceptibility as


$$\begin{array}{c} u_{i}^{\text{info}}=\frac{{Var}_{i}[P]}{{Var}_{\max}}, \end{array}$$ (16)

where ${Var}_{\max}$ is the variance when $k_{i}^{\text{obs}}=0$ (no known neighbor states), ensuring $0<u_{i}^{\text{info}}<1$.

This formulation captures the ambiguity in risk assessment created by incomplete information—especially relevant in privacy-preserving, decentralized tracing systems where neighborhood states are only partially visible. By explicitly accounting for unobserved contacts, the model links epidemiological dynamics to information constraints in digital health technologies.

### 2.4. Experience dependent mechanism

Human behaviour reflects a mix of model-free and model-based reinforcement learning [26], combining accumulated experience with forward-looking considerations. The Experience-Weighted Attraction (EWA) framework captures this integration [57], bringing together reinforcement learning [58] and belief learning [59] by assigning equal weight to realized and forgone payoffs. In our setting, forgone payoffs are inferred from neighbours’ average infection rates because individuals do not observe others’ outcomes directly [60]. Agents evaluate payoffs over the past *m* cycles, with memory decaying at a rate determined by perceived epidemiological risk. EWA thus describes how individuals merge past experience with beliefs about others’ behaviour. Its empirical support [27] makes it a suitable, cognitively grounded foundation for modelling social adaptation and norm compliance.

At the end of each season, agents evaluate the payoffs associated with the two available actions—vaccinate or not vaccinate:


$$\begin{array}{c} \begin{array}{c} \Pi_{i}^{Unvac}(t)=\left\{ \begin{array}{cc} 1-c_{I}E_{i}[P](t), & \text{if vaccinated},\\ 1-c_{I}{\hat{I}}_{i}(t), & \text{if unvaccinated}, \end{array}\right.\\ \Pi_{i}^{Vac}(t)=1-c_{V}, \end{array} \end{array}$$ (17)

where $c_{V}$ and $c_{I}$ denote the costs of vaccination and infection, respectively.

**Memory decay:** Let ${\hat{\pi }}_{i}^{Vac}(t)$ and ${\hat{\pi }}_{i}^{Unvac}(t)$ represent the average payoffs of the two actions over the past *m* seasons. Since the payoff from vaccination is constant,


$$\begin{array}{c} {\hat{\pi }}_{i}^{Vac}(t)=\Pi_{i}^{Vac}=1-c_{V}. \end{array}$$ (18)

For the unvaccinated option, memory decays over time depending on the agent’s emotional state. When perceived safety $S_{i}(t)$ is low, fear increases attention to recent outcomes, causing older experiences to be discounted more strongly:


$$\begin{array}{c} {\hat{\pi }}_{i}^{Unvac}(t)=\sum_{j=0}^{m}\frac{(S_{i}(t){)}^{j}}{\sum_{n=0}^{m}(S_{i}(t){)}^{n}}\Pi_{i}^{Unvac}(t-j), \end{array}$$ (19)

where $S_{i}(t)$ is the safety parameter defined previously.

The asymmetry in memory treatment reflects a simplifying modeling choice. In the baseline specification, the payoff from vaccination is represented as a fixed direct cost, whereas the payoff from non-vaccination depends on uncertain infection outcomes and is therefore more naturally subject to recency-weighted updating under changing perceived risk [13,36]. For this reason, memory decay is applied to the non-vaccination payoff while the vaccination payoff is kept fixed. This is not the only plausible specification. In settings where vaccine efficacy, side effects, booster requirements, or perceived burden vary over time, a symmetric decay structure could also be justified. The present asymmetry should therefore be seen as a tractable baseline assumption rather than as a general behavioral claim.

**Regret:** Anticipated regret is a key driver of preventive behavior. Empirical studies on influenza and COVID-19 vaccination show that regret and worry mediate the link between perceived risk and uptake, shaping both immediate and future choices [10,13,61,62]. Regret thus operates as an experience-weighted feedback mechanism—past outcomes influence future intentions [36].

To formalize this, we employ the Loomes–Sugden (LS) model of regret [9,63], which introduces an additive regret–rejoice term to utility without conflating it with risk aversion. The payoff adjusted for regret is


$$\begin{array}{c} \pi_{Vac}={\hat{\pi }}_{Vac}-R({\hat{\pi }}_{Unvac}-{\hat{\pi }}_{Vac}), \end{array}$$ (20)

and symmetrically for $\pi_{Unvac}$, where


$$\begin{array}{c} R(x)=\left\{ \begin{array}{cc} \eta_{1}x^{\eta_{2}}, & x>0,\\ 0, & x\leq 0, \end{array}\right. \end{array}$$ (21)

with $\eta_{1}$ and $\eta_{2}$ denoting the strength and curvature of regret. When an inferior option is chosen, the resulting utility is reduced by the regret of not selecting the better alternative.

The LS model provides a parsimonious, empirically supported account of emotional feedback. It captures how anticipated regret promotes immediate vaccination, whereas experienced regret shapes future intentions [10,30]. Unlike Prospect Theory, which emphasizes perceptual biases such as loss aversion and probability weighting [64], the LS formulation isolates regret’s motivational role as a corrective, counterfactual mechanism—well suited to repeated, feedback-driven settings like seasonal vaccination [29].

We employ the LS model because it reproduces a key empirical regularity: anticipated regret predicts vaccination more reliably than perceived risk, while experienced regret updates subsequent behavior. It does so with minimal parameters and clear interpretability, directly linking emotional self-evaluation to learning and adaptation.

The timing of regret in the present framework deserves brief clarification. The regret term enters current choice in an anticipatory sense, because agents evaluate each action partly through comparison with the forgone alternative [9,29]. At the same time, these evaluations are shaped by realized outcomes from previous seasons, since the payoff terms entering the regret-adjusted comparison are updated through experience [30,36]. The model therefore links prospective and retrospective elements: regret is applied at the moment of choice, but its content is informed by earlier epidemic outcomes and their behavioral consequences.

Although *normative regret* (e.g., guilt or social disapproval) could be modeled similarly, evidence indicates these emotions operate mainly ex post, reinforcing self-correction and social alignment rather than foresight [30,65]. Accordingly, we restrict regret here to material outcomes and introduce normative regret, i.e., social disapproval, in the next section on norm dynamics.

### 2.5. Norm dynamics

To integrate social norms into the Experience-Weighted Attraction (EWA) framework, we separate material payoffs from normative dynamics. Empirical research identifies three complementary influences—descriptive norms (what others do), injunctive norms (what others approve of), and personal norms (one’s moral standard) [3,37]. These operate through distinct cognitive pathways and evolve on different timescales. Longitudinal evidence from the Swiss COVID-19 vaccination campaign shows that media tone primarily shaped injunctive norms, whereas descriptive norms closely tracked observed vaccination behavior [38]. Furthermore, online experiments suggest that when social information is limited or unreliable, personal norms best predict behavior [39]. With such empirical evidence in mind, our model tracks the joint evolution of $y_{i}$, ${\tilde{x}}_{i}$, and ${\tilde{y}}_{i}$, capturing both social learning and moral feedback.

Agents are boundedly rational [66]: they infer others’ attitudes from observed actions [67,68], balancing the avoidance of social disapproval with the need for internal coherence. The three normative variables are updated after each decision cycle, distinguishing between the discrete vaccination action and the continuous intention $x_{i}$—unlike [28], where actions were modeled as continuous.

The dynamics of personal norms ($y_{i}$), injunctive expectations (${\tilde{y}}_{i}$), and descriptive expectations (${\tilde{x}}_{i}$) follow a DeGroot-type formulation [28]. The corresponding terms represent cognitive dissonance (reducing action–attitude mismatch), social projection (assuming similarity to others [69]), logical consistency between beliefs and observed actions [70], and peer or authority conformity [71]. Dissonance aligns $x_{i}$ and $y_{i}$, ensuring that attitudes adjust toward past behavior [72].

For interpretability, we normalize these equations:


$$\begin{array}{c} \begin{array}{c} y_{i}^{\prime }=y_{i}+\xi_{i}^{1}[\hat{C_{i}^{11}}x_{i}+\hat{C_{i}^{12}}X_{i}+\hat{C_{i}^{13}}G_{i}^{1}-y_{i}]\\ {\tilde{y}}_{i}^{\prime }={\tilde{y}}_{i}+\xi_{i}^{2}[\hat{C_{i}^{21}}y_{i}+\hat{C_{i}^{22}}X_{i}+\hat{C_{i}^{23}}G_{i}^{2}-{\tilde{y}}_{i}]\\ {\tilde{x}}_{i}^{\prime }={\tilde{x}}_{i}+\xi_{i}^{3}[\hat{C_{i}^{31}}{\tilde{y}}_{i}+\hat{C_{i}^{32}}X_{i}+\hat{C_{i}^{33}}G_{i}^{3}-{\tilde{x}}_{i}], \end{array} \end{array}$$ (22)

where $\xi_{i}^{q}=\sum_{j}C_{i}^{qj}$ denotes each variable’s updating rate (*q* = 1,2,3), and $\hat{C_{i}^{qj}}=C_{i}^{qj}/\sum_{k}C_{i}^{qk}$ ensures $\sum_{j}\hat{C_{i}^{qj}}=1$.

**Influence weights and trust:** We initially neglect external authorities by setting $\hat{C_{i}^{q3}}=0$, implying $\hat{C_{i}^{q2}}=1-\hat{C_{i}^{q1}}$. When external interventions are included, they are uniform across agents: $\hat{C_{i}^{q3}}=\gamma_{q}$. Each agent’s update thus depends on internal consistency ($x_{i}$, $y_{i}$, ${\tilde{y}}_{i}$) and social influence ($X_{i}$). We interpret $\hat{C_{i}^{q1}}$ as the self-consistency weight and $1-\hat{C_{i}^{q1}}$ as the social influence weight. These coefficients are coupled to the epidemic environment through the trust factor $\theta_{i}^{Col}(t)$, derived earlier:


$$\begin{array}{c} \hat{C_{i}^{q1}}=1-\theta_{i}^{Col}(t),\;\hat{C_{i}^{q2}}=\theta_{i}^{Col}(t), \end{array}$$

where $\theta_{i}^{Col}(t)$ depends on the change-detector and consensus functions defined previously. Fig 2 schematically illustrates these relationships.

Unless stated otherwise, we set $\xi_{i}^{1}=0.01$, $\xi_{i}^{2}=0.1$, and $\xi_{i}^{3}=1$, reflecting that personal norms evolve more slowly than injunctive or descriptive expectations ($\xi^{3}>\xi^{2}>\xi^{1}$). Results remain robust as long as this hierarchy is preserved. This hierarchy is experimentally justified as evidence shows that personal norms remain stable and predictive even when empirical and normative expectations vary, suggesting that personal moral evaluations are less context-dependent than descriptive or injunctive cues [39]. Future extensions could introduce state-dependent $\xi_{i}^{q}$, potentially yielding qualitative transitions similar to those reported in [73].

It is also useful to distinguish between endogenous and exogenous elements of the model. Endogenous quantities include perceived safety, informational uncertainty, peer stability, local consensus, adaptive decision weights, vaccination intentions, actions, and normative states, all of which evolve during the simulation. By contrast, the functional forms linking these quantities, together with baseline parameter values, initialization rules, and network-generation choices, are exogenous modeling assumptions. This distinction matters for interpretation: the results arise from endogenous feedback operating within a specified behavioral architecture, rather than from directly imposing aggregate outcomes from the outset [20,21].

Table 1 summarizes the main model parameters, their interpretation, and the rationale for their baseline values. The model is not calibrated to a specific epidemic dataset. Instead, parameter values are chosen to fall within empirically plausible ranges, to preserve interpretability, and to allow systematic examination of how the coupled behavioral mechanisms shape collective outcomes [10,11,26,29,40]. Where a parameter has a substantively important effect on results, this dependence is examined explicitly in the Results section.

**Table 1:** Main model parameters, baseline values, and interpretation.

Parameter	Meaning	Baseline	Basis for choice
*N*	Number of agents	500	Standard baseline size for simulation experiments
β	Infection transmission rate	varied	Main epidemiological control parameter
μ	Recovery rate	1	Normalization of epidemic timescale
$c_{V}$	Vaccination cost	1	Utility normalization
$c_{I}$	Infection cost	0.1	Baseline relative cost in payoff comparisons
*m*	Memory length	4	Baseline value; varied to assess robustness
$\eta_{1}$	Regret strength	varied	Main behavioral control parameter
$\eta_{2}$	Regret curvature / greediness	varied	Main behavioral control parameter
$k_{rat}$	Choice stochasticity / inverse precision	varied	Governs sensitivity to utility differences
*z*	Fraction of observed neighbors	varied	Controls informational uncertainty
$u_{i}^{intr}$	Intrinsic uncertainty	0.1	Baseline low uncertainty level
$\xi_{1}$	Updating rate of personal norms	0.01	Assumed slowest normative adjustment
$\xi_{2}$	Updating rate of injunctive expectations	0.1	Intermediate normative adjustment
$\xi_{3}$	Updating rate of descriptive expectations	1	Assumed fastest normative adjustment
γ	Intervention strength	1.0	Baseline intervention intensity
*G*	Intervention target value	0.5	Benchmark target in intervention experiments
ϵ	Equilibrium tolerance	0.01	Stopping criterion
$n_{sim}$	Number of SIR realizations per season	1000	Stable estimation of infection-related quantities

## 3. Results

We ran 1,000 simulations of the Susceptible–Infected–Recovered (SIR) process on a network of *N* = 500 nodes, using the event-driven algorithm of [35] in each season. Vaccination and infection costs were set to $c_{V}=1$ and $c_{I}=0.1$, with intrinsic susceptibility uncertainty $u_{i}^{intr}=0.1$ (results were robust for nearby values). Unless otherwise stated, memory length was *m* = 4 and transmission rate β=6. Initial personal, descriptive, and injunctive norms were drawn independently from uniform distributions. Each run started with a single infected agent and no initial vaccinations, and continued until equilibrium, defined as a vaccination-rate change below 0.01 during the last 50 of a maximum 200 seasons. Reported outcomes are medians with interquartile ranges.

Robustness checks on Erdős–Rényi and scale-free networks with average degree ⟨k⟩=6 showed variations in vaccination and infection levels below 5%, indicating that the main qualitative patterns depend more on clustering and degree heterogeneity than on one specific topology. A detailed analysis of network topology is beyond the scope of the present paper.

Parameter choices were guided by empirical evidence from influenza and COVID-19 vaccination studies, as well as by work on regret and probabilistic choice. Anticipated regret is a strong predictor of vaccination: a one-standard-deviation increase typically raises intention by 10–25% [10,11,13]. Setting the regret-sensitivity coefficient to $\eta_{1}\approx 1$ on a normalized utility scale reproduces this order of magnitude, while $\eta_{2}\in [0.5,1]$ captures moderate self-serving bias, in line with experimental estimates of regret concavity [29,30]. The choice-precision parameter $k_{rat}$ governs stochasticity in logit choice. Values between 0.1 and 1 correspond to precision levels $\lambda =1/k_{rat}\in [1,10]$, consistent with quantal-response estimates from coordination and strategic games [26,40]. Within this range, agents select the higher-utility option with approximately 65–90% probability, which is compatible with the observed variability of preventive behavior. Parameters were not fitted to data, but chosen within empirically grounded bounds in order to capture realistic, probabilistic, and norm-sensitive dynamics. Table 1 summarizes the main baseline parameters and their interpretation.

### 3.1. Social norms evolution

**Fig 3:**
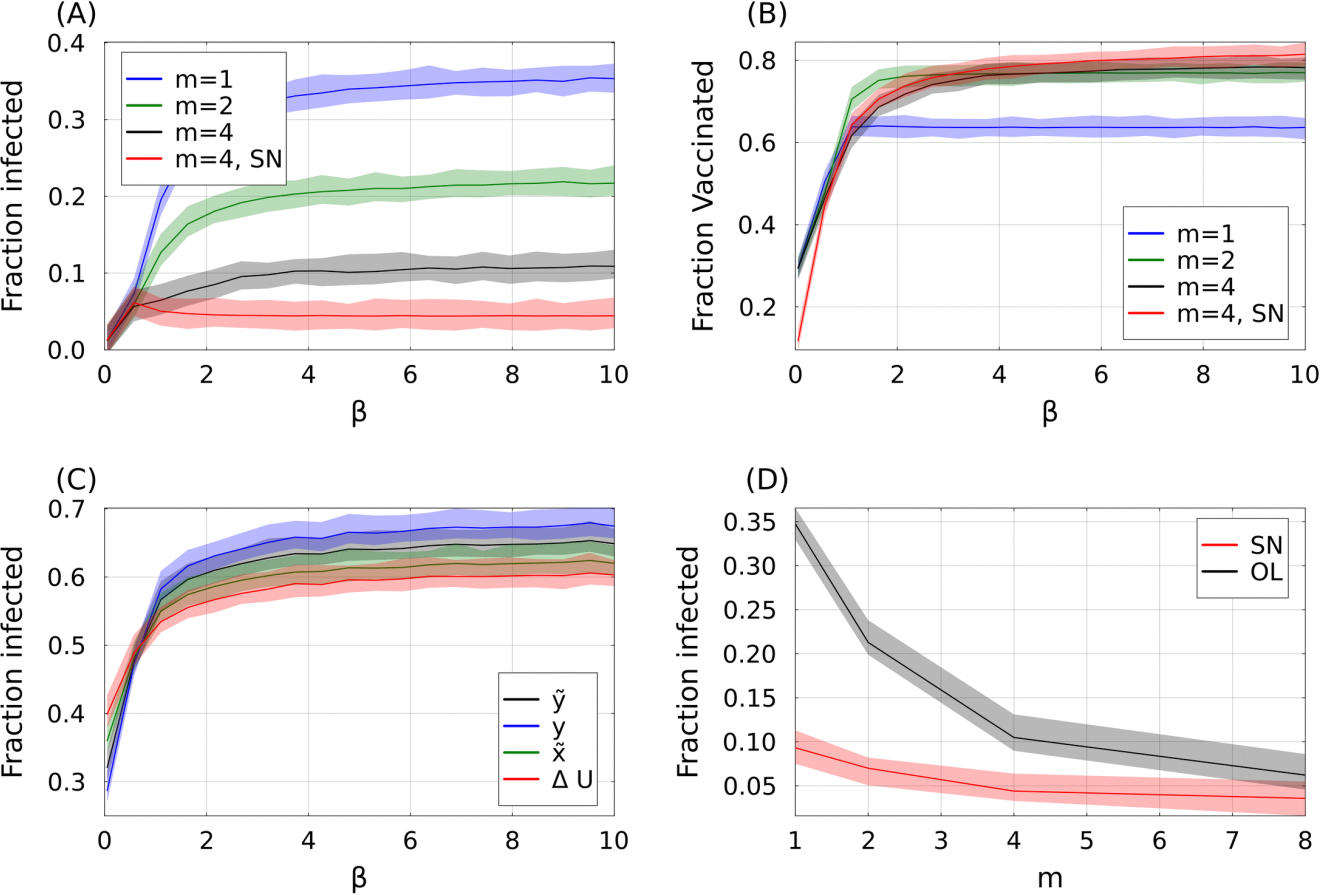
Behavioral and epidemiological effects of memory and social norms. **(A)** Fraction of infected individuals vs. infectivity rate β. Without social norms, increasing β raises infection levels. Comparing memory lengths *m* = 1 (blue), *m* = 2 (green), and *m* = 4 (black), longer memory—hence greater informational depth—consistently lowers infections. When agents also consider perceived norms (red), infections drop further for the same memory, indicating that normative feedback stabilizes preventive behavior. **(B)** Fraction of vaccinated individuals vs. infectivity rate β. Vaccination coverage rises with both β and memory but quickly saturates. Including social norms (*m* = 4) does not increase mean vaccination relative to learning-only agents yet improves its spatial distribution. Norms therefore enhance coordination and reduce clustering rather than overall uptake ($k_{rat}=0.1$). **(C)** Evolution of social norms vs. infectivity rate β. The intention $\Delta U_{i}$ and the three norm components remain misaligned because they adapt at different rates: empirical expectations adjust fastest, while personal and injunctive norms evolve more slowly ($k_{rat}=0.1$). **(D)** Fraction of infected individuals vs. memory length. With only learning (OL), infections decline sharply as memory increases, reflecting improved decision accuracy. When social norms (SN) are included, infection rates become less sensitive to memory, showing that normative feedback substitutes for experience. Social influence thus reduces reliance on individual memory to sustain cooperative vaccination.

Fig 3A and 3B show the joint evolution of epidemic and behavioral dynamics under varying cognitive and normative conditions. In Fig 3, infection levels rise with infectivity β, but both longer memory and social norms markedly reduce outbreak size. Agents with extended memory (*m* = 4 vs. 1–2) incorporate more payoff history, vaccinate more consistently, and face smaller epidemics. Adding social norms (red line) further suppresses infections for the same memory length, even when average vaccination coverage changes little. Norms therefore improve the *distribution* rather than the *level* of vaccination—reducing clustering and stabilizing uptake. Similar average vaccination rates do not necessarily imply similar epidemic outcomes. Even when mean uptake differs only modestly across memory conditions, the timing and distribution of vaccination across the contact network can change substantially. Longer memory stabilizes vaccination decisions and can reduce unfavorable temporal fluctuations or local clustering of unprotected individuals. As a result, epidemic size may fall even when the average vaccination level changes only slightly. This interpretation is consistent with a broader literature showing that epidemic risk depends not only on aggregate vaccination coverage, but also on how susceptibility is distributed across social and spatial structures: clustering of susceptible individuals can substantially increase outbreak probability even when overall immunity remains high [74,75]. More recent work also shows that vaccination timing can affect epidemic dynamics independently of average coverage, including cases in which poorly timed vaccination produces worse subsequent peaks [76].

Fig 3C depicts the values of personal norms (*y*), empirical expectations ($\tilde{x}$), injunctive expectations ($\tilde{y}$), and behavioral intentions (*x*) at equilibrium as a function of infectivity β. These variables remain distinct because they evolve at different speeds: empirical expectations react most rapidly to observed behavior, injunctive norms adjust more slowly through social feedback, and personal norms change gradually through reflection. This temporal hierarchy prevents convergence and captures the asymmetry described by [23,37], where descriptive norms follow short-term cues while injunctive norms act as slower moral anchors stabilizing behavior under changing risk.

**Fig 4:**
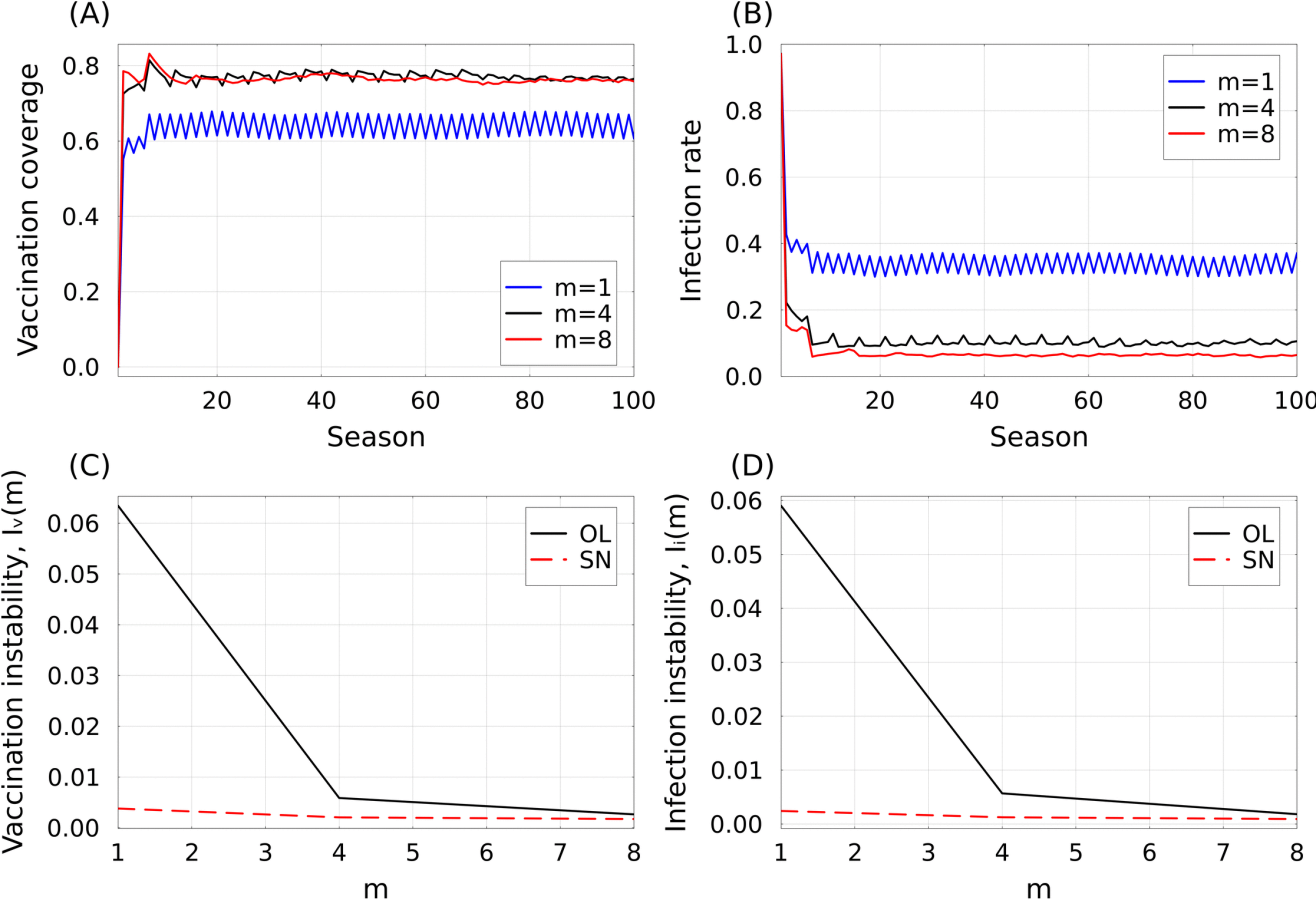
Temporal stability of vaccination and infection under different memory lengths. Panels A and B show the season-by-season evolution of ensemble-mean vaccination coverage and infection rate in the learning-only specification for *m* = 1, *m* = 4, and *m* = 8. Panels C and D summarize the corresponding temporal-instability measures defined in Eqs. (23)–(24) for both the learning-only (OL) and social-norm (SN) specifications. Under learning only, increasing memory sharply reduces both vaccination and infection instability, especially between *m* = 1 and *m* = 4, indicating that longer recall stabilizes adaptive behavior and dampens short-run switching. Under social norms, instability is already low and much less sensitive to memory, showing that normative feedback partly substitutes for individual experience as a source of behavioral stabilization.

**Fig 5:**
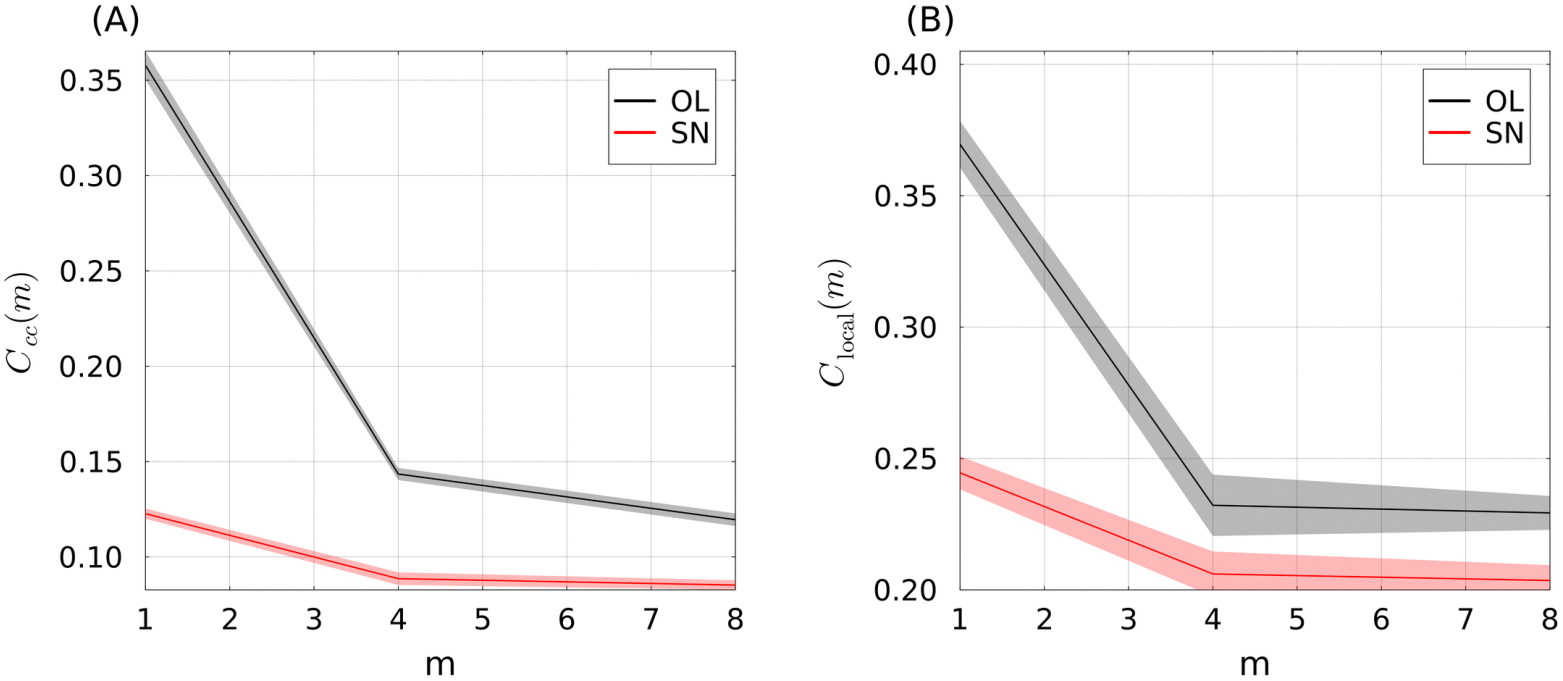
Memory reduces clustering of susceptible individuals on the physical layer. Panel A shows the size of the largest connected component of unvaccinated individuals, normalized by population size, corresponding to Eq. (26). Panel B shows the mean unvaccinated-neighbor share around unvaccinated nodes, corresponding to Eq. (25). In the learning-only specification, both measures fall sharply as memory increases, indicating that longer recall breaks up connected pockets of susceptible individuals and reduces local concentration of non-vaccinators. Under social norms, both clustering measures are already lower and become only weakly dependent on memory. This shows that memory and norms improve epidemic outcomes not only by changing average behavior, but also by redistributing protection in a way that makes large outbreaks less likely.

Finally, Fig 3D illustrates how memory length influences vaccination dynamics. Longer recall produces steadier vaccination and smaller outbreaks, as extended memory dampens short-term fluctuations. When social norms are included, the marginal effect of memory weakens—normative feedback already promotes behavioral stability. Beyond moderate memory (m≥4), gains plateau, indicating that limited recall suffices to sustain cooperative vaccination. To make this mechanism more explicit, Figs 4 and 5 below examine two complementary diagnostics: the temporal stability of vaccination and infection across seasons, and the network distribution of unvaccinated individuals on the physical contact layer. Together, these diagnostics show that the effect of memory is not exhausted by average uptake alone. Memory changes how steadily protection is maintained over time and how strongly unvaccinated individuals remain clustered in epidemiologically vulnerable parts of the network.

#### 3.1.1. Temporal stability and network distribution under memory.

The interpretation above can be made more explicit by examining how memory affects both the temporal stability of the dynamics and the spatial organization of susceptibility on the physical contact network. Let ${\bar{v}}_{t}(m)$ and ${\bar{i}}_{t}(m)$ denote, respectively, the ensemble-mean vaccination coverage and infection rate at season *t* for memory length *m*. Here, the infection rate at season *t* refers to *t*he mean fraction of infected individuals during that season, averaged across simulation runs for *t*he corresponding memory condition. To summarize temporal instability after a burn-in period starting at season $t_{B}$, we define


$$\begin{array}{c} {ℐ}_{v}(m)=\frac{1}{T-t_{B}}\sum_{t=t_{B}}^{T-1}\left|{\bar{v}}_{t+1}(m)-{\bar{v}}_{t}(m)\right|, \end{array}$$ (23)

and


$$\begin{array}{c} {ℐ}_{i}(m)=\frac{1}{T-t_{B}}\sum_{t=t_{B}}^{T-1}\left|{\bar{i}}_{t+1}(m)-{\bar{i}}_{t}(m)\right|. \end{array}$$ (24)

These quantities measure the average season-to-season change in vaccination and infection, so larger values correspond to more unstable dynamics. To improve readability, the displayed time series are lightly smoothed using a short moving average, but the qualitative ordering across memory conditions is robust.

Fig 4 shows that memory has a pronounced stabilizing effect in the learning-only specification. In panels A and B, the *m* = 1 case displays both lower vaccination and substantially higher infection than the *m* = 4 and *m* = 8 cases. More importantly, the lower panels show that the temporal-instability measures ${ℐ}_{v}(m)$ and ${ℐ}_{i}(m)$ fall sharply as memory increases. The largest change occurs between *m* = 1 and *m* = 4, while the difference between *m* = 4 and *m* = 8 is much smaller, indicating diminishing returns beyond moderate memory. This pattern clarifies that longer memory does not merely shift the average level of behavior; it also makes vaccination and epidemic outcomes more stable across seasons.

The same figure also shows why the role of memory becomes weaker once social norms are introduced. Under the full social-norm specification, the instability measures remain low for all values of *m* considered. In other words, normative feedback already stabilizes behavior, so increasing individual recall provides less additional benefit. This result strengthens the interpretation of Fig 3D: social norms partly substitute for memory by reducing the sensitivity of collective outcomes to short-run fluctuations in individual experience.

Temporal stabilization is only part of the explanation. To examine how memory affects the distribution of protection across the physical layer, let $U_{t}(m)$ denote the set of unvaccinated individuals at season *t* and let ${𝒩}_{i}$ be the set of physical neighbors of node *i*. We consider *t*wo complementary diagnostics. First, we define the local unvaccinated-neighbor share


$$\begin{array}{c} {𝒞}_{local}(m)=\frac{1}{\left|U_{t}(m)\right|}\sum_{i\in U_{t}(m)}\left(\frac{1}{\left|{𝒩}_{i}\right|}\sum_{j\in {𝒩}_{i}}1_{\left\{j\in U_{t}(m)\right\}}\right), \end{array}$$ (25)

which measures the average fraction of unvaccinated neighbors surrounding an unvaccinated node. Second, we define


$$\begin{array}{c} {𝒞}_{cc}(m)=\frac{\left|LCC(U_{t}(m))\right|}{N}, \end{array}$$ (26)

where $LCC(U_{t}(m))$ is the largest connected component of the subgraph induced by unvaccinated nodes on the physical layer. The first diagnostic is local, the second global, but both capture whether susceptibility remains concentrated in epidemiologically dangerous clusters.

Fig 5 shows that both clustering measures decline markedly with memory in the learning-only case. For *m* = 1, unvaccinated individuals are much more likely to remain connected to one another, both locally and through a large connected component. Increasing memory substantially reduces these concentrations of susceptibility, especially between *m* = 1 and *m* = 4. Under social norms, by contrast, both ${𝒞}_{local}(m)$ and ${𝒞}_{cc}(m)$ are already lower and become only weakly dependent on memory. This means that social norms not only reduce instability, but also break up connected pockets of unprotected individuals.

Taken together, Figs 4 and 5 clarify the mechanism underlying the memory result. Memory affects epidemic outcomes through at least two channels: it stabilizes vaccination behavior across seasons, and it reduces the formation of clustered susceptible regions on the physical network. The first channel helps explain why outbreaks are smaller even when average uptake does not change dramatically; the second explains why similar aggregate vaccination levels can still generate different epidemic burdens. These results therefore support and sharpen the interpretation already suggested by Fig 3: what matters is not only how many agents vaccinate on average, but also how steadily and how favorably protection is distributed over time and across the contact structure.

#### 3.1.2. Systematic decomposition and bounded rationality.

**Fig 6:**
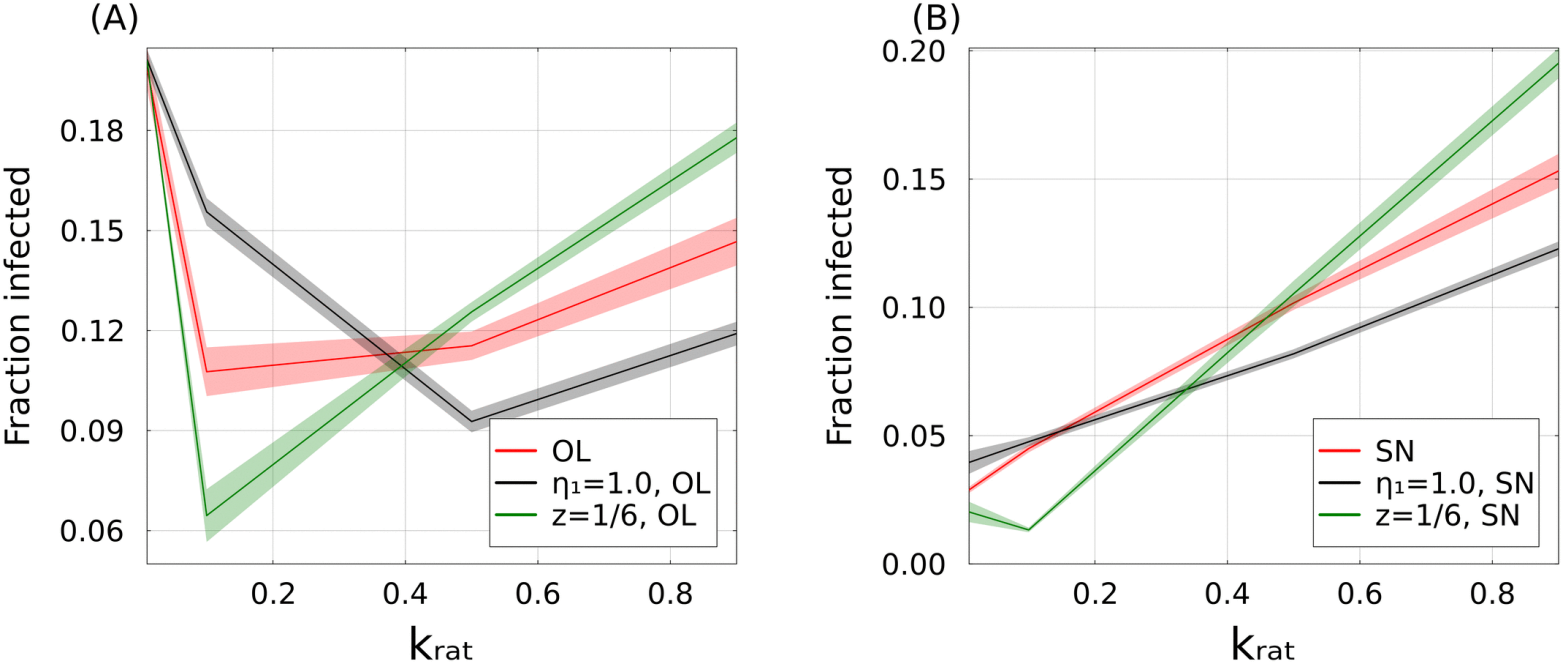
Systematic decomposition of choice-precision effects across model variants. The figure compares the final fraction of infected individuals as a function of the choice-precision parameter $k_{rat}$ under two model specifications. Left: learning-only baseline (OL). Right: full model with social norms (SN). In each panel, the baseline case is compared with a specification including regret ($\eta_{1}=1.0$) and a specification with increased uncertainty (*z* = 1/6). In the OL baseline, epidemic size is non-monotonic in $k_{rat}$, with infections minimized at intermediate values. In the SN baseline, by contrast, infection levels increase more steadily with $k_{rat}$, so that the intermediate optimum observed in OL is no longer present in the same form. Regret affects the two environments differently: in OL it worsens outcomes at low $k_{rat}$ but improves them at intermediate and high $k_{rat}$, whereas in SN it reduces infection levels across the full range shown. Increased uncertainty has a strongly conditional effect in both cases: it produces very low infection levels when $k_{rat}$ is small, but becomes increasingly detrimental as $k_{rat}$ rises. Taken together, the two panels show that regret and uncertainty do not have uniform effects of their own, but instead reshape epidemic outcomes through their interaction with choice precision and with the presence or absence of normative feedback.

To clarify which qualitative patterns arise from adaptive learning alone and which depend on the interaction between learning, regret, uncertainty, and normative feedback, we examine the effect of the choice-precision parameter $k_{rat}$ across reduced and extended model variants. Fig 6 compares the final epidemic burden in a baseline learning-only specification (OL) and in the full specification with social norms (SN), with separate curves showing the baseline case, the case with regret, and the case with increased uncertainty. For each case, the figure also shows how the relationship changes when regret is introduced (here illustrated for $\eta_{1}=1.0$) and when uncertainty is increased (here illustrated for *z* = 1/6). A more detailed treatment of regret and uncertainty is provided in the subsequent subsections; here the aim is to identify, at the level of this decomposition, how these mechanisms reshape the effect of choice precision.

Fig 6 shows first that the dependence of epidemic size on $k_{rat}$ differs qualitatively across the baseline OL and SN specifications. In the learning-only case, the baseline curve is clearly non-monotonic, with infections minimized at intermediate values of $k_{rat}$. This indicates that the best collective outcomes arise when agents are neither too noisy nor too rigid in the way they translate utility differences into action. When $k_{rat}$ is large, choice becomes too stochastic and agents fail to respond consistently to utility differences. When $k_{rat}$ becomes very small, behavior becomes overly deterministic, reducing exploration and making adaptation to changing conditions more difficult. Intermediate values therefore provide the most favorable balance between responsiveness and flexibility [18,26,40].

In the full model with social norms, the baseline dependence on $k_{rat}$ is different. Under SN, infection levels increase more steadily with $k_{rat}$, and the intermediate optimum observed in OL is no longer present in the same form. Instead, the best outcomes occur at low values of $k_{rat}$, while greater decision noise progressively worsens epidemic performance. This indicates that normative feedback changes not only the level of infections but also the shape of the effect of choice precision. In particular, social norms appear to stabilize behavior sufficiently that highly deterministic choice is no longer associated with the same detrimental rigidity observed in the learning-only case, whereas high decision noise remains costly.

The additional curves in Fig 6 show that regret and uncertainty further reshape these bounded-rationality patterns. In the OL case, introducing regret ($\eta_{1}=1.0$) raises infection levels at low $k_{rat}$ relative to the baseline, but lowers them at intermediate and high $k_{rat}$, thereby making the interior minimum more pronounced. For the parameter values shown here, regret therefore has a dual effect: when decisions are already highly deterministic it appears to amplify rigidity, whereas at noisier levels of choice it can play a corrective role and improve outcomes. Under SN, by contrast, the regret curve lies below the baseline across the full range of $k_{rat}$ shown, suggesting that normative feedback turns regret into a more consistently beneficial force by channeling it through a more stable social environment.

Uncertainty, illustrated here by the case *z* = 1/6, has an even more sharply context-dependent effect. In both OL and SN, increased uncertainty yields the lowest infection levels at very small values of $k_{rat}$, but the corresponding curve rises steeply as $k_{rat}$ increases and eventually becomes worse than the baseline. Thus, for the parameter values shown in Fig 6, uncertainty is beneficial only when agents respond rather precisely to utility differences; once decision-making becomes noisier, uncertainty substantially degrades collective outcomes. This pattern is especially strong in the SN case, where uncertainty produces the steepest increase in infections as $k_{rat}$ grows. In this sense, normative feedback does not fully offset the destabilizing effect of uncertainty when choice precision is low.

Taken together, Fig 6 isolates several distinct points. First, the non-monotonic effect of $k_{rat}$ is already an emergent property of adaptive learning itself rather than something imposed by the norm component. Second, social norms modify this baseline relationship by stabilizing deterministic choice and reshaping the effect of behavioral stochasticity. Third, the effects of regret and uncertainty are themselves conditional on the broader behavioral environment: regret can be either destabilizing or corrective in OL but becomes more uniformly beneficial under SN, while uncertainty can improve outcomes under sufficiently precise choice but becomes strongly harmful as decision noise increases. The more systematic analysis of these two mechanisms is presented in the following subsections, but the decomposition already makes clear that their epidemiological consequences depend critically on how they interact with choice precision and with the presence or absence of normative feedback. Accordingly, the role of choice precision in the model should not be summarized by a single universal optimum: its epidemiological consequences depend on whether decisions are guided only by adaptive learning or also by normative feedback.

### 3.2. Regret

**Fig 7:**
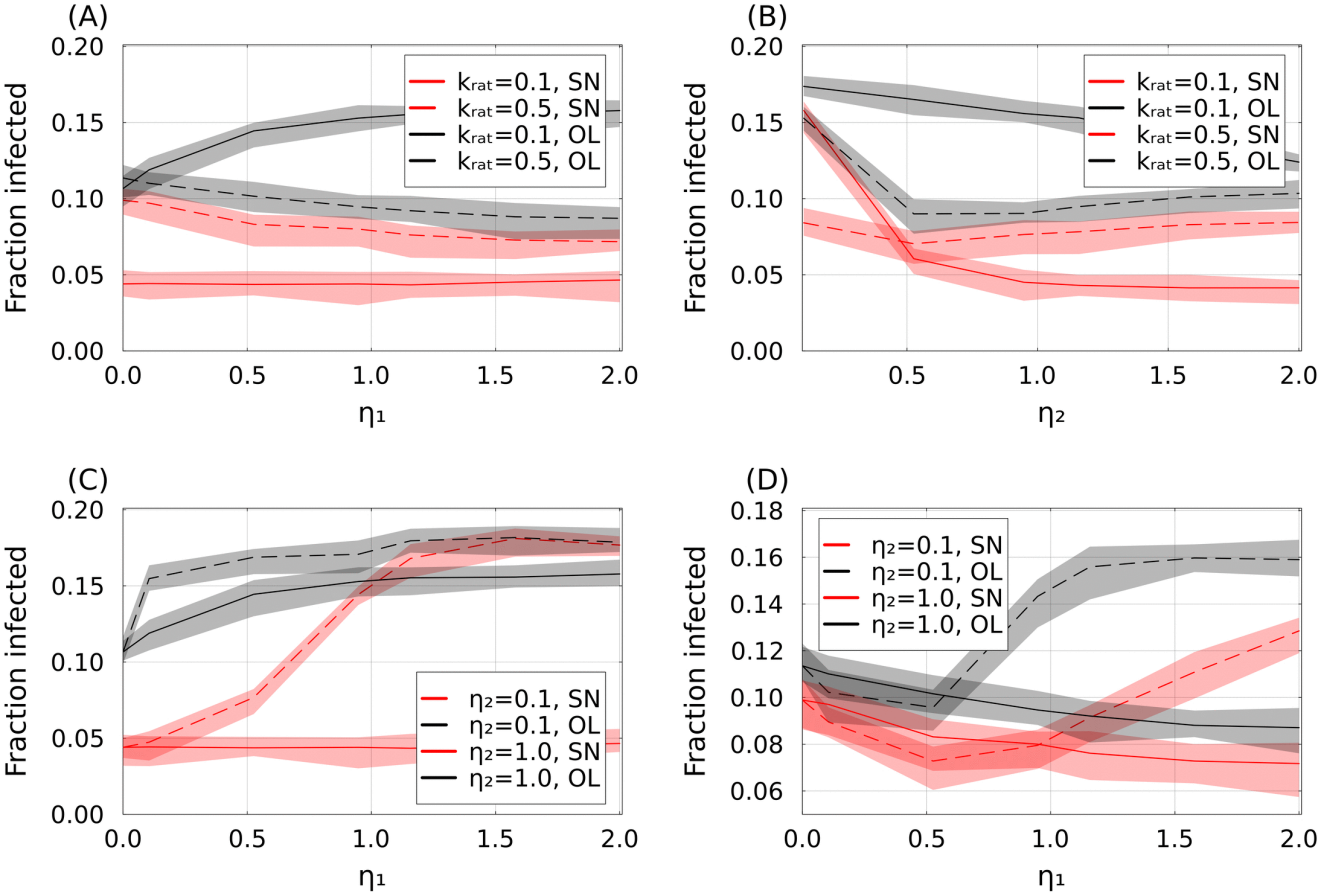
Behavioral impact of regret and greediness parameters. (A) Fraction of infected individuals vs. regret strength $\eta_{1}$. With greediness fixed at $\eta_{2}=1.0$, high-precision agents ($k_{rat}=0.1$) perform worse as regret increases without norms, while outcomes remain more stable when norms are included. For lower-precision agents ($k_{rat}=0.5$), intermediate regret improves results in both cases, suggesting that regret can support adaptive exploration when choice is noisier. (B) Fraction of infected individuals vs. greediness $\eta_{2}$. At fixed regret strength $\eta_{1}=1.0$, reducing greediness benefits high-precision agents in both norm and no-norm settings, with larger gains under normative influence. For lower-precision agents, the effect is non-monotonic: both extreme greed (low $\eta_{2}$) and weak sensitivity (high $\eta_{2}$) worsen outcomes, whereas intermediate values yield lower infection levels. (C) Fraction of infected individuals across $\eta_{1}$ for $k_{rat}=0.1$. (D) Same for $k_{rat}=0.5$. For high-precision, high-greed agents ($k_{rat}=0.1$, $\eta_{2}=0.1$), increasing $\eta_{1}$ reduces the difference between norm and no-norm conditions, indicating less adaptive use of social information. For lower-precision agents ($k_{rat}=0.5$), an intermediate $\eta_{1}$ minimizes infections, suggesting that moderate regret is associated with more favorable collective outcomes.

Fig 7A and 7B show how the regret parameters—the strength of regret $\eta_{1}$ and the degree of greediness $\eta_{2}$—affect epidemic outcomes. In Fig 7A, the influence of regret varies with agents’ choice precision. For high-precision agents ($k_{rat}=0.1$), increasing $\eta_{1}$ worsens outcomes without norms, as excessive sensitivity produces less adaptive updating and more unstable learning dynamics. With social norms present, this instability is dampened because normative feedback moderates overcorrection. For lower-precision agents ($k_{rat}=0.5$), intermediate regret improves performance in both settings, reducing infections through more adaptive exploration. The right panel of Fig 7B further shows that lower greediness (higher $\eta_{2}$) improves collective outcomes, especially under normative influence, suggesting that prosocial restraint complements regret-based adaptation. Overall, regret acts as a double-edged mechanism: moderate levels promote coordination and stability, whereas excessive regret destabilizes decision-making.

Fig 7C and 7D maps infection levels across the $\eta_{1}-\eta_{2}$ space. For highly rational and greedy agents ($k_{rat}=0.1$, $\eta_{2}=0.1$), stronger regret cancels the benefit of norms, as the resulting dynamics become less adaptive and less responsive to new information and social feedback. For lower-precision agents ($k_{rat}=0.5$), an intermediate $\eta_{1}$ minimizes infections, showing that regret-driven feedback can stabilize collective behavior when cognitive precision is limited. This non-monotonic pattern mirrors empirical evidence that moderate anticipated regret fosters preventive action, while excessive regret or greed weakens cooperation.

These dynamics align with longitudinal findings on influenza vaccination: individuals who initially avoided vaccination but later experienced regret were more likely to vaccinate the following year, whereas those who regretted vaccinating were less likely to repeat it [10]. The model reproduces this asymmetry—moderate regret promotes adaptive correction, while excessive regret in payoff-driven agents induces overreaction. Consistent with regret theory [77], strong regret aversion can lead to counterproductive behavior. Thus, regret acts as both corrective and fragile—adaptive when moderate, distortionary when extreme—linking affective learning, choice precision, and social norms.

### 3.3. Uncertainty

**Fig 8:**
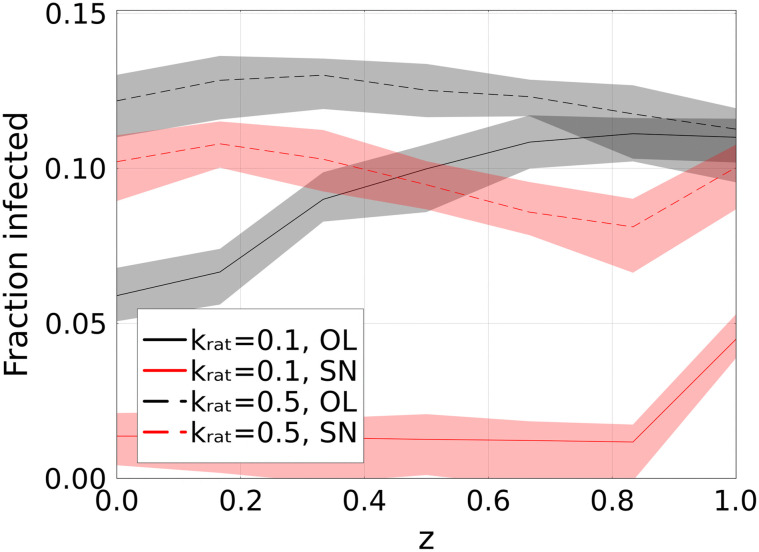
Fraction of infected individuals vs. percentage of observed neighbors *z.* For high-precision agents ($k_{rat}=0.1$) relying only on individual learning, reduced information about neighbors’ states promotes caution, raising vaccination and reducing outbreaks. In contrast, for lower-precision agents ($k_{rat}=0.5$), limited information hampers learning and increases infections. When social norms are included, high-precision agents again achieve lower infection levels than when acting independently. For lower-precision agents, moderate information scarcity can be beneficial by limiting overreaction to noisy cues, but excessive uncertainty ultimately worsens outcomes.

Fig 8 illustrates how informational uncertainty, expressed as the percentage of observed neighbors *z*, shapes epidemic outcomes under different levels of choice precision. For high-precision agents ($k_{rat}=0.1$) relying solely on personal experience, reduced information (lower *z*) induces precautionary behavior and greater vaccination, resulting in smaller outbreaks. In contrast, lower-precision agents ($k_{rat}=0.5$) fare worse under limited information: uncertainty magnifies decision noise and raises infection levels.

Including social norms alters this relationship. High-precision agents achieve substantially lower infection rates across all *z*, as normative feedback compensates for missing epidemiological information. For lower-precision agents, moderate uncertainty can even be advantageous, curbing overreliance on unreliable cues, though excessive opacity once again disrupts coordination. Overall, the effect of uncertainty is non-monotonic: partial ignorance can foster caution and cooperation when supported by reasoning or social guidance, but too much uncertainty undermines collective stability.

### 3.4. Interventions

**Fig 9:**
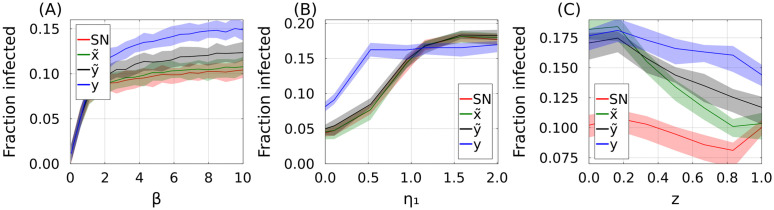
Intervention effects through different normative channels. (A) Fraction of infected individuals vs. infectivity rate β (*G* = 0.5, γ=1.0). Interventions acting through personal norms *y* are most effective, indicating their stronger behavioral leverage relative to social norms. Empirical expectations $\tilde{x}$ respond least, reflecting their dependence on observed collective behavior. (B) Fraction of infected individuals vs. regret strength $\eta_{1}$ under interventions (*G* = 0.5, γ=1.0). For high-precision agents ($k_{rat}=0.1$), effectiveness shifts with rising $\eta_{1}$: at low regret sensitivity, interventions through personal norms are strongest, whereas at higher $\eta_{1}$ interventions through social norms become relatively more effective. (C) Fraction of infected individuals vs. percentage of observed neighbors *z* under interventions (*G* = 0.5, γ=1.0). For lower-precision agents ($k_{rat}=0.5$), interventions through personal norms are most effective when information is complete. Under high uncertainty (low *z*), this ordering reverses, with social norms becoming the more influential intervention channel.

We examine how external interventions targeting different normative dimensions affect epidemic outcomes. Each intervention has strength γ=1.0, driving the targeted norm toward a desired value *G* = 0.5, below the level reached without intervention (see Fig 3B). Fig 9A shows the resulting infection fraction as a function of the infectivity rate β.

Effects vary across normative domains. Interventions on personal norms *y* produce the largest reduction in infections, indicating that self-endorsed moral standards are both flexible and strongly linked to behavior. Targeting injunctive expectations $\tilde{y}$ yields moderate gains, while interventions on empirical expectations $\tilde{x}$—reflecting perceived peer behavior—have the weakest impact, as descriptive norms adjust only through observed collective behavior and resist external influence.

These results are in agreement with empirical work suggesting that personal norms are both highly predictive of behavior and relatively malleable through targeted interventions [39]. Furthermore, they also align with experimental evidence that injunctive norms are more adaptable than descriptive ones [8,38,68]. Overall, interventions are most effective when shaping internal moral evaluations rather than perceptions of others’ actions, emphasizing the importance of belief formation in public health communication.

#### 3.4.1. Regret.

Fig 9B illustrates how interventions modulating regret strength $\eta_{1}$ affect epidemic outcomes across normative targets. Each intervention has intensity γ=1.0 and target value *G* = 0.5. For high-precision agents ($k_{rat}=0.1$), a crossover in effectiveness emerges: at low intervention levels, targeting personal norms *y* yields the strongest reduction in infections, whereas stronger interventions acting on social norms—particularly empirical ($\tilde{x}$) and injunctive ($\tilde{y}$) expectations—become relatively more effective.

This shift suggests that cognitively precise agents internalize social feedback more effectively. As choice becomes more precise, altering collective expectations appears to promote coordinated vaccination more strongly, while individual-focused interventions lose relative influence once social coupling becomes more behaviorally salient. Overall, the success of regret-based strategies depends jointly on emotional sensitivity $\eta_{1}$ and the normative dimension targeted. Leveraging collective regret signals can thus align individual heuristics with collective welfare under bounded rationality.

#### 3.4.2. Uncertainty.

Fig 9C illustrates how intervention effectiveness varies with informational uncertainty, measured by the percentage of observed neighbors *z*, across different normative targets. For lower-precision agents ($k_{rat}=0.5$), a clear reversal emerges as information becomes scarce. With full visibility, interventions on personal norms *y* are most effective, since well-informed individuals rely primarily on internal moral evaluation. As uncertainty increases (lower *z*), social norms gain influence, with injunctive expectations $\tilde{y}$ and descriptive expectations $\tilde{x}$ eventually surpassing personal norms in effectiveness.

This transition indicates that under limited visibility, individuals rely more heavily on socially mediated cues than on self-guided judgment. Hence, uncertainty shifts the optimal intervention channel from personal to social norms, revealing that the informational environment critically determines behavioral leverage.

Across parameter sweeps, three mechanisms consistently sustain coordination: (i) intermediate bounded rationality balances exploration and learning, (ii) moderate regret promotes adaptive correction, and (iii) uncertainty redistributes normative influence, transferring effectiveness from self-oriented to socially mediated pathways.

## 4. Conclusions

This study develops a behavioral epidemic model that combines regret, uncertainty, and social norms within a single agent-based framework. The main contribution is not simply to place several psychologically motivated mechanisms in the same formal structure, but to examine the collective consequences of their interaction. By embedding regret-adjusted evaluation, uncertainty-dependent weighting, and the coevolution of personal, descriptive, and injunctive norms within repeated epidemic decision-making, the model identifies qualitative patterns that are difficult to infer when these mechanisms are studied separately. These include non-monotonic effects in the learning-only baseline, the reshaping of these effects under normative feedback, and reversals in the relative effectiveness of interventions under informational scarcity [18,20,21].

The simulations reveal four main interaction-based findings. First, choice precision has context-dependent effects. Here, $k_{rat}$ denotes the stochasticity of the logit choice rule: lower values correspond to more precise, utility-sensitive decisions, whereas higher values correspond to noisier decisions. In the learning-only baseline, epidemic outcomes are most favorable at intermediate values of $k_{rat}$. At this level, agents are responsive enough to payoff differences to avoid random behavior, but still flexible enough to adapt when conditions change. This result is consistent with the broader interpretation of probabilistic choice models, where some behavioral noise can preserve adaptability while excessive noise weakens responsiveness to incentives [26,40]. When social norms are introduced, this relationship is reshaped because normative feedback anchors choices in more persistent personal, descriptive, and injunctive expectations. Highly precise choice therefore no longer produces the same loss of adaptability observed in the learning-only case. Instead, infections are lowest at low values of $k_{rat}$, while higher values increasingly undermine the stabilizing role of norms by weakening agents’ responsiveness to the utility and normative signals that support vaccination.

Second, regret can either improve or destabilize collective outcomes. Moderate regret supports adaptive correction, especially when decisions are noisy, whereas excessive regret can destabilize behavior when agents respond too rigidly to perceived payoff differences. This pattern is consistent with empirical evidence showing that anticipated regret can increase vaccination intentions, while regret theory also predicts that strong counterfactual sensitivity may produce counterproductive choices when individuals overreact to forgone outcomes [10,11,29].

Third, uncertainty changes the channels through which social norms affect behavior. Rather than simply reducing cooperation, it shifts the relative influence of personal, descriptive, and injunctive norms. This helps explain why different intervention targets become more or less effective as information becomes scarce. The result is consistent with evidence that incomplete information and low confidence increase reliance on social cues, and that descriptive and injunctive messages can have different effects on preventive or risk-related behavior [7,8,14].

Fourth, memory and norms improve epidemic outcomes through stabilization and spatial redistribution, not only through average vaccination uptake. Longer memory and normative feedback stabilize vaccination over time and reduce connected clusters of susceptible individuals on the physical contact network. This mechanism is consistent with epidemiological evidence showing that outbreak risk depends not only on aggregate vaccination coverage, but also on the timing of vaccination and on how susceptibility is distributed across contact structures [74, 75, 76].

The model also clarifies why the effectiveness of norm-based interventions depends on the informational environment. When information is abundant, targeting personal norms is most effective, because moral self-consistency provides a strong guide for behavior [1,2]. Under uncertainty, however, socially mediated cues become more influential, so interventions targeting injunctive or descriptive expectations can become comparatively more effective. This pattern is consistent with experimental evidence showing that informational context affects whether internal or social motives sustain cooperation [7,25]. The greater flexibility of injunctive norms relative to descriptive norms [8,38,68] suggests that moral communication and institutional signaling may be especially important when direct behavioral feedback is limited.

Methodologically, the Regret–Uncertainty model illustrates how agent-based modeling can bridge psychological realism and epidemiological dynamics. By embedding cognitive, emotional, and normative heterogeneity into decision rules, it extends previous vaccination ABMs [18,22–24] and generalizes experience-weighted attraction learning to include affective feedback and endogenous norm evolution. Unlike imitation models that treat norms as external, this framework internalizes moral and descriptive expectations, enabling targeted interventions and linking behavioral economics with computational epidemiology.

Beyond vaccination, the model contributes to the broader study of norm change and collective behavior [1,21] by showing how emotions and trust can dynamically reweight moral and pragmatic motives. Future work could extend this framework to coevolving networks, where interaction structures and norms adapt jointly [28,70], and to cross-cultural settings in which conformity and sanctioning differ systematically [78]. Combining behavioral experiments and online field data with simulations would help test and refine these mechanisms in real-world contexts [2,21].

### 4.1. Model limitations

The model is intended as a stylized, mechanism-based account of how regret, uncertainty, and norms interact in repeated epidemic decision-making. Its conclusions should therefore be interpreted at that level, and several limitations help define the scope of that interpretation.

Within the behavioral architecture specified here, the analysis is internally coherent. At the same time, some results depend on modeling choices such as the adaptive weighting scheme, the asymmetric memory structure, and the treatment of uncertainty under partial observability. The analysis explores the behavior of the model across broad parameter ranges and briefly notes that qualitatively similar patterns were obtained under alternative network structures, although these comparisons are not developed into a full robustness analysis. Some conclusions may therefore remain sensitive to the specific functional forms and structural assumptions adopted here.

The model is designed to represent regret, uncertainty, trust, and norm-guided behavior in a tractable way, not to provide a direct measurement model of psychological constructs. In particular, the functions linking fear, uncertainty, peer stability, and local consensus to adaptive decision weights are informed by existing literature rather than estimated directly from data [14,15,38]. Likewise, the baseline specification treats the vaccination payoff as fixed while allowing the non-vaccination payoff to be updated asymmetrically through experience [13,36]. These choices improve interpretability but they do not exhaust all plausible behavioral formulations.

The framework is not calibrated to a specific real-world epidemic or vaccination campaign. Its purpose is explanatory rather than narrowly predictive. Moreover, the network structures, initial conditions, and intervention targets are synthetic and therefore cannot capture the full institutional and cultural complexity of real vaccination environments [33,34]. The results should therefore be read as qualitative insights into the interaction of regret, uncertainty, and norm dynamics, rather than as precise quantitative forecasts for a particular case. Future work could strengthen external validity through empirical calibration, richer behavioral measurement, alternative updating rules, and more realistic network and policy environments.
